# A Strategy to Delay the Development of Cisplatin Resistance by Maintaining a Certain Amount of Cisplatin-Sensitive Cells

**DOI:** 10.1038/s41598-017-00422-2

**Published:** 2017-03-27

**Authors:** Guihua Duan, Qianyuan Tang, Hongli Yan, Lijuan Xie, Yun Wang, Xi Emily Zheng, Yuzheng Zhuge, Shanshan Shen, Bin Zhang, Xiaoqi Zhang, Jun Wang, Wei Wang, Xiaoping Zou

**Affiliations:** 1 Department of Gastroenterology, Drum Tower Hospital, Medical School of Nanjing University, Nanjing, 210008 China; 2 Department of Gastroenterology, The First People’s Hospital of Yunnan Province, The Affiliated Hospital of Kunming University of Science and Technology, Kunming, 650032 China; 3 National Laboratory of Solid State Microstructure, Department of Physics, Nanjing University, Nanjing, 210093 China; 4 Collaborative Innovation Center of Advanced Microstructures, Nanjing University, Nanjing, 210093 China; 5 Department of Laboratory Medicine, Changhai Hospital, Second Military Medical University, Shanghai, 200433 China; 6 Department of Special Medical Treatment, First Affiliated Hospital of Kunming Medical University, Kunming, 650332 China

## Abstract

Cisplatin (ddp), which is commonly employed in the treatment of many advanced cancers, often results in initial therapeutic success; however, rapid progression of ddp-resistant cells remains the main reason for treatment failure. Facd with such a problem, we investigated the fitness differences between ddp-sensitive and ddp-resistant cell lines. We found that the growth of ddp-resistant cells was significantly slower than that of sensitive cells due to elevated ROS levels, which suggested that the ddp resistance mechanisms may have negative impacts on the growth of resistant cells. Furthermore, we observed that, when mixed with ddp-sensitive cells, ddp-resistant cells failed to compete, and the growth of ddp-resistant cells could therefore be suppressed by treatment *in vivo*. We propose a mathematical model parameterized based on *in vivo* experiments to describe the allometric growth of tumors consisting of two competing subclones. According to our model, a quantitative strategy with a variant drug-dosing interval is proposed to control tumor growth. Taking advantage of intratumoral competition, our strategy with appropriate dosing intervals could remarkably delay the development of ddp resistance and prolong overall survival. Maintaining a certain number of ddp-sensitive cells rather than eradicating the tumor with continuous treatment is feasible for future tumor treatment.

## Introduction

Cytotoxic treatment is one major method for inhibiting tumors. Such treatments may at first successfully control tumor growth, but the tumor can eventually evolve to become drug-resistant and rapidly regrow. For example, platinum-based drugs, particularly cisplatin (ddp), are commonly employed in the treatment of many advanced cancers^1^. Similar to other treatments, ddp often leads to initial therapeutic success, but resistant subclones eventually expand. During these processes, intratumor heterogeneity is one of the essential determinants of such evolution, and there is increasing evidence indicating the presence of resistant subclones prior to the initiation of therapy^2–4^. During disease progression, different subclones evolve over time under microenvironmental or selective pressure following the principles of *Darwinian* evolution^5–8^. For tumors treated with platinum-based drugs, such evolution may become the major impediment to clinical treatment and could lead to the expansion of drug-resistant subclones^6, 9–12^.

For platinum-based drugs^13^, the therapy-induced promotion of drug resistance suggests that drug-resistant cells might exhibit a fitness deficit in the absence of the drug since drug resistance mechanisms require the consumption of additional resources for proliferation, as suggested by previous theories^14^. However, the fitness differences between ddp-sensitive and ddp-resistant cells have not been examined previously, and the relationship between the mechanism of ddp resistance and fitness differences is still unclear. In the cytoplasm, the interaction between ddp and reduced glutathione (GSH) has the potential to disrupt the redox balance, and reactive oxygen species (ROS) can facilitate ddp-induced DNA damage or directly trigger mitochondrial outer membrane permeabilization (MOMP)^1^. These findings suggest that ROS homeostasis may play a crucial role in both ddp resistance and cell fitness. Maintaining ROS homeostasis is crucial for cell proliferation and survival^15^. Therefore, ROS homeostasis may also have an important impact on the growth of ddp-resistant cells.

In a tumor that consists of multiple subclones, the fitness differences of the diverse subclones give rise to competition between them^16^. When drug-resistant cells belong to the less fit subclones, taking advantage of such competition may be a practical way to retard the progression of drug resistance in tumors. Thus, Gatenby *et al*., suggested that patient survival time might be prolonged by exploiting the competition between doxorubicin-sensitive and doxorubicin-resistant cells based on computational models^17^. In their work, the fitness differences between doxorubicin-resistant and doxorubicin-sensitive cells relied on verapamil and 2-deoxyglucose to emphasize the cost of resistance according to *in vitro* experiments, which was insufficient to explain the competition between drug-resistant cells and drug-sensitive cells *in vivo*. Direct experimental evidence and applications related to the growth competition between drug-sensitive cells and drug-resistant cells are currently limited, and were the targets of the present study.

In our work, we first focused on the fitness of ddp-sensitive and ddp-resistant cells, which were characterized based on their growth rates. We found that the growth rate of ddp-resistant cells was slower than that of ddp-sensitive cells *in vitro* due to reduced proliferation and an increased apoptosis rate. We also confirmed that the growth of ddp-resistant cells was substantially slower than that of sensitive cells *in vivo*. We subsequently observed that the growth disadvantage of ddp-resistant cells resulted from an elevated ROS level and could be rescued by GSH. Based on such differences in fitness between ddp-sensitive cells and ddp-resistant cells, systems involving the mixture of two subclones could better resemble the growth of real tumors. Therefore, we constructed a xenograft model and mathematical model for tumors consisting of two competing subclones with different fitness levels. Our xenograft model showed that when ddp dosing was performed with appropriate intervals and frequencies, the size of tumors containing both ddp-sensitive cells and ddp-resistant cells could still be controlled at a certain level (100–200 mm^3^) after 40 days, while the size of tumors containing only ddp-resistant cells could not be controlled (exceeding 500 mm^3^). Furthermore, our mathematical model, which was parameterized based on *in vivo* experiments, confirmed that such a strategy could lead to both long survival (5-fold longer than under continuous dosing) and a lower tumor burden. Our strategy could delay the development of ddp resistance by taking advantage of the competitive relationships between ddp-sensitive cells and ddp-resistant cells rather than by eradicating ddp-sensitive cells. Such a strategy would be practically for future tumor treatment without changing the medicines utilized.

## Results

### The growth of ddp-resistant cells is slower than that of sensitive cells in vitro

**Figure 1:**
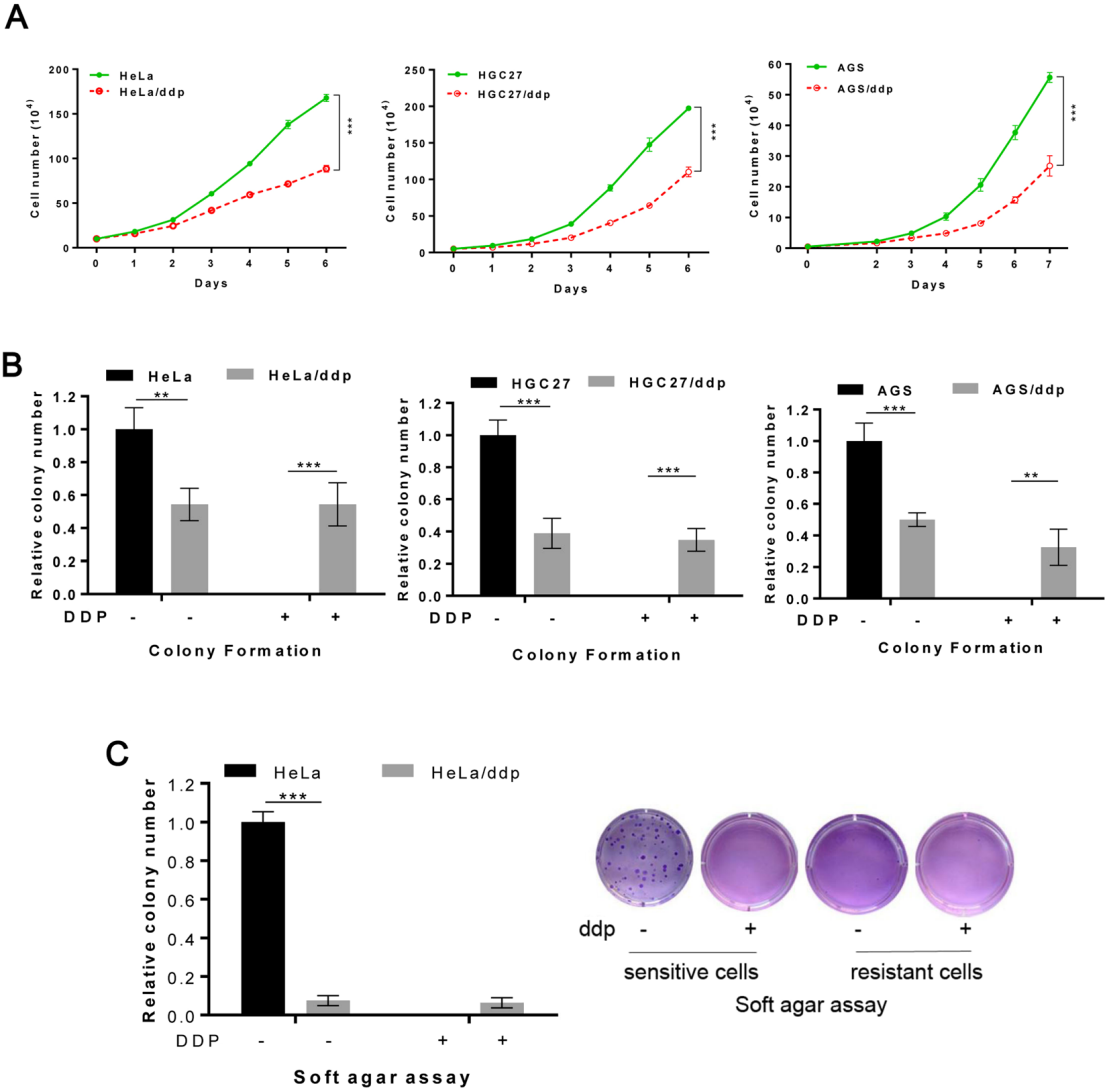
The growth of resistant cells is markedly slower than that of sensitive cells *in vitro*. (**A**) Growth of HeLa, HeLa/ddp, HGC27, HGC27/ddp, AGS and AGS/ddp cells; the medium was exchanged with normal medium every day. (**B**) Relative clonogenic growth of HeLa, HeLa/ddp, HGC27, HGC27/ddp, AGS and AGS/ddp cells under the indicated conditions; ddp (0.5–0.7 μg/ml) was added to the medium on the following day. (**C**) Soft agar assay of HeLa and HeLa/ddp cells under the indicated conditions. Cisplatin (0.5 μg/ml) was added to the medium on the following day. The error bars represent the s.d. of triplicate wells of a representative experiment.

**Figure 2:**
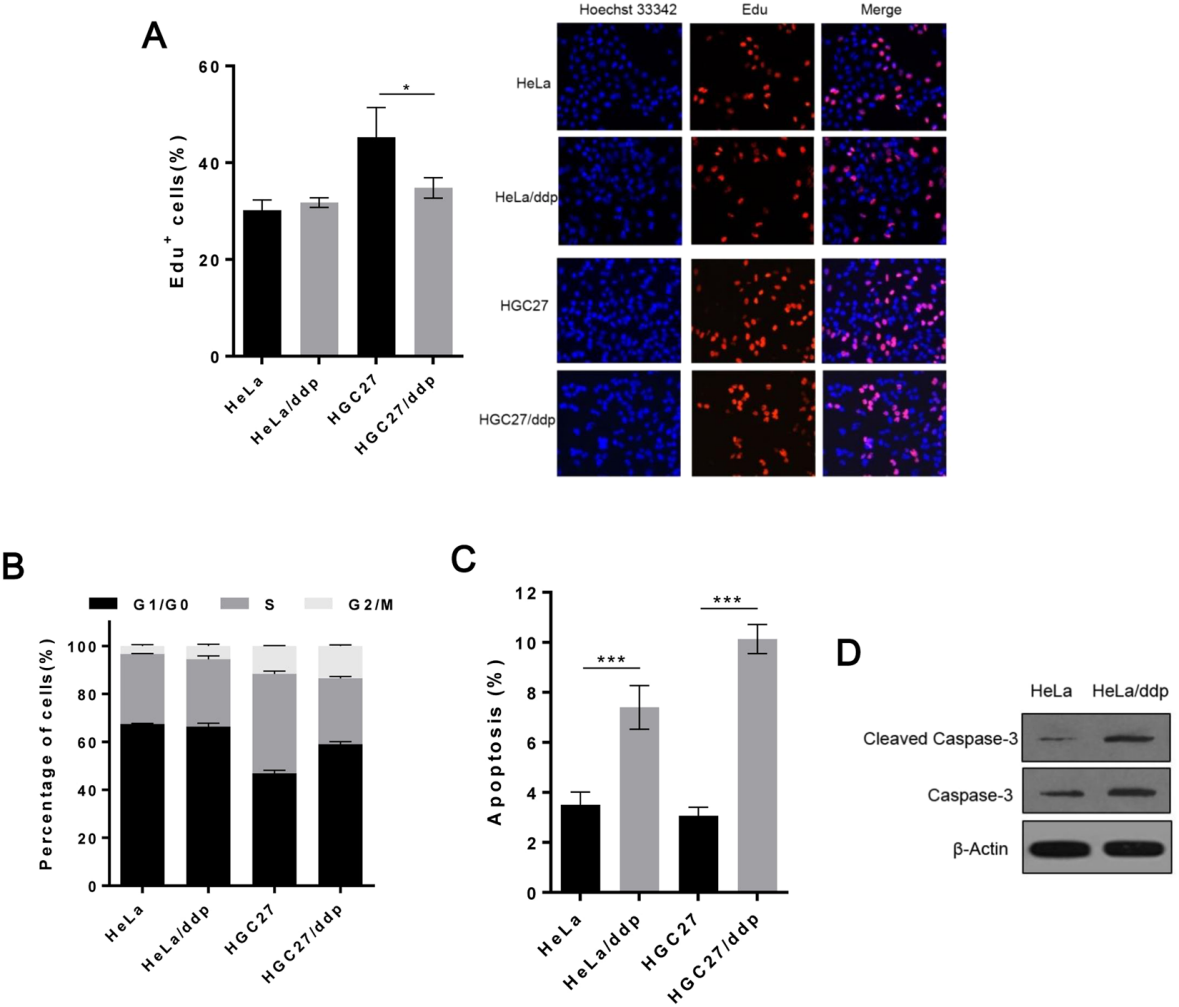
The apoptosis rate is increased in ddp-resistant cells. (**A**) EdU assay of HeLa, HeLa/ddp, HGC27 and HGC27/ddp cells. The medium was replaced with normal medium on the following day. The EdU assay was performed 48 h after medium replacement. (**B**) Cell cycle analysis of HeLa, HeLa/ddp, HGC27 and HGC27/ddp cells. The medium was replaced with normal medium on the following day. The cell cycle distribution was analyzed 48 h after medium replacement. (**C**) Apoptosis of HeLa, HeLa/ddp, HGC27 and HGC27/ddp cells under the indicated conditions. The medium was replaced on the following day with normal medium. Apoptosis rate was analyzed 72 h after medium replacement. (**D**) Western blot analysis of cleaved caspase-3, and caspase-3 protein expression in HeLa and HeLa/ddp cells using different antibodies, β-Actin was used as a loading control. The error bars represent the s.d. of triplicate wells of a representative experiment.

First, we compared the growth abilities of these two types of cell lines *in vitro*. The growth rates of resistant cell lines were slower than those of sensitive cell lines (Fig. 1A). Fewer colonies were observed for the resistant cells than for the sensitive cells in the colony-formation assay. However, the clonogenic growth of resistant cells was not affected by ddp, whereas the growth of sensitive cells was completely inhibited (Fig. 1B and Supplementary Fig. S2A). In particular, the anchorage-independent growth of resistant cells was considerably slower than that of sensitive cells as determined in soft agar assays (Fig. 1C). These results indicated that the growth of resistant cells was apparently slower than that of sensitive cells in the absence of the drug. Furthermore, the EdU-incorporation assay and cell cycle analysis indicated that there were no differences in DNA synthesis or the cell cycle in the presence of abundant nutrition in HeLa and HeLa/ddp cell lines, but G1 arrest and reduced DNA synthesis were observed in HGC27/ddp cells compared with that in HGC27 cells (Fig. 2A and B). In addition, apoptosis was increased in both HeLa/ddp and HGC27/ddp cells (Fig. 2C), and an increased propensity to undergo apoptotic cell death was demonstrated by the enhanced expression of cleaved caspase-3 in resistant cells (Fig. 2D).

### The growth disadvantage of ddp-resistant cells is rescued by adding reduced glutathione (GSH)

**Figure 3:**
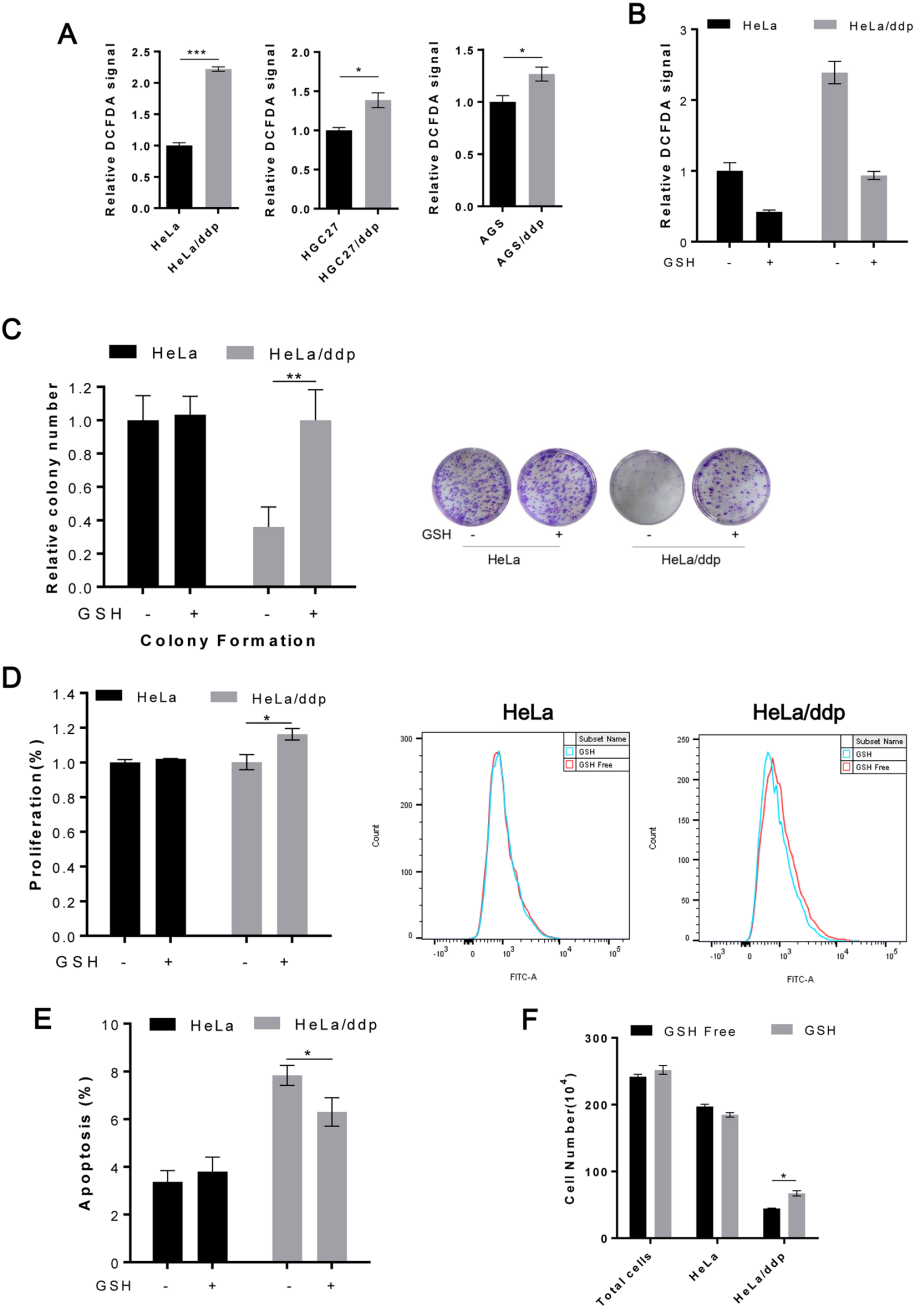
The growth disadvantage of ddp-resistant cells is rescued by decreasing ROS levels. (**A**) Relative ROS levels in HeLa, HeLa/ddp, HGC27, HGC27/ddp, AGS and AGS/ddp cells. (**B**) Relative ROS levels in HeLa and HeLa/ddp cells under the indicated conditions. (**C**) Relative clonogenic growth of HeLa and HeLa/ddp cells under the indicated conditions. GSH (4 mM) was added to the medium on the following day. (**D**) Relative proliferation of HeLa and HeLa/ddp cells under the indicated conditions. Proliferation was determined through CFSE assays after six days. (**E**) Relative apoptosis of HeLa and HeLa/ddp cells under the indicated conditions. (**F**) Growth of HeLa and HeLa/ddp cells when cultured together; HeLa cells were labeled with CFSE; HeLa cells and HeLa/ddp cells were plated in 6-well plates at 2 × 10^5^ cells per well in an equal ratio; GSH (4 mM) was added to the medium on the following day. Total cell numbers were counted with a Hand-held Automated Cell Counter, and the ratio of sensitive cells was analyzed via flow cytometry. The error bars represent the s.d. of triplicate wells of a representative experiment.

Next, we observed that the basic ROS level was elevated in ddp-resistant cells (Fig. 3A), and we therefore further evaluated the impact of GSH on the growth of both cell lines. GSH could decrease ROS levels in both cell lines (Fig. 3B) and significantly increase the colony-formation rate in resistant cells but not in sensitive cells (Fig. 3C and Supplementary Fig. S2B). Further investigation indicated that GSH could promote proliferation and decrease apoptosis in resistant cells, whereas sensitive cells were not affected (Fig. 3D and E). We also found that GSH could increase the growth of resistant cells when resistant cells were mixed with sensitive cells (Fig. 3F); although the growth of sensitive cells appeared to be suppressed under this condition, the difference was not significant. Collectively, these results suggested that although ddp-resistant cells manifested a strong ability to survive toxic treatment, increased ROS level also became a proliferation burden, which was not observed in sensitive cells.

### Ddp-resistant cells show poor adaptability in vivo

**Figure 4:**
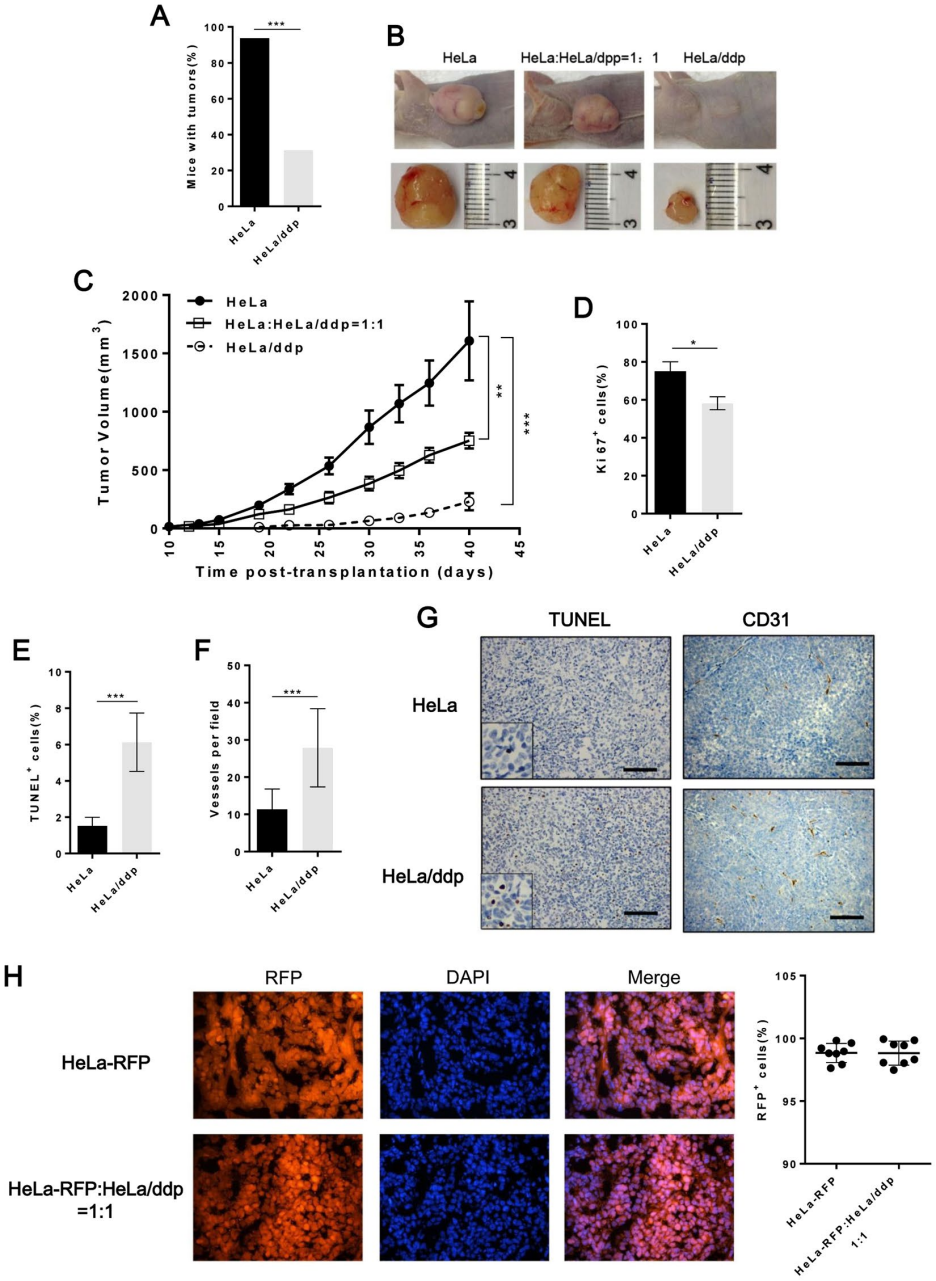
Resistant cells show poorer adaptability than sensitive cells *in vivo*. (**A**) HeLa and HeLa/ddp cells were implanted and monitored for tumor establishment over a period of 30 days (n = 16 per group). (**B**) Representative images of tumors. (**C**) Tumor growth kinetics (n = 16 per group); the error bars indicate s.e.m. (**D**) Quantification of anti-Ki67 immunohistochemical staining in HeLa and HeLa/ddp tumors (n = 3 mice per group). The error bars indicate s.d. (**E**) Quantification of TUNEL^+^ cells per field (n = 3 mice per group). The error bars indicate s.d. (**F**) Quantification of the average number of CD31^+^ vessels per field (n = 3 mice per group). The error bars indicate s.d. (**G**) Representative images of the staining indicated in Fig. **E** and **F**. (**H**) Quantification of the average number of RFP^+^ cells per field; each dot represents an individual tumor. Tumor cells were isolated when the animal was euthanized, 40 days post-transplantation. The error bars indicate s.d.

To verify whether the results observed in cultured cells were relevant to tumorigenesis *in vivo*, we evaluated the growth adaptability of both cell lines in mice. Initially, we noted that significantly fewer ddp-resistant than ddp-sensitive cells grew in the mice (Fig. 4A), and the growth of resistant tumors was remarkably slower than that of sensitive tumors (Fig. 4B and C). Further investigation indicated that resistant tumor cells proliferated more slowly than sensitive tumor cells (Fig. 4D and Supplementary Fig. S3A). In resistant tumors, 6% of the cells were apoptotic cells compared with 1.5% in sensitive tumors (Fig. 4E and G). Furthermore, we investigated changes in the tumor microenvironment. Resistant tumors displayed a higher intratumoral micro-vascular density than sensitive tumors, indicating that resistant tumors required more vessels to supply nutrients and oxygen (Fig. 4F and G). To account for competition dynamics between resistant and sensitive cell lines, we also established a group of tumors consisting of resistant cells and RFP-tagged sensitive cells (Supplementary Fig. S3B) at an initial ratio of 1:1. There was no difference in the number of RFP-positive cells between the sensitive cell groups and mixed groups when the tumors were harvested, indicating that the growth of resistant cells was completely inhibited by sensitive cells (Fig. 4H). Together, these data suggested that the resistant cell lines presented a significant fitness deficit *in vivo*, and their growth was completely suppressed when they coexisted with sensitive cells.

### The development of ddp-resistant cells is delayed by maintaining a certain number of ddp-sensitive cells during treatment in vivo

**Figure 5:**
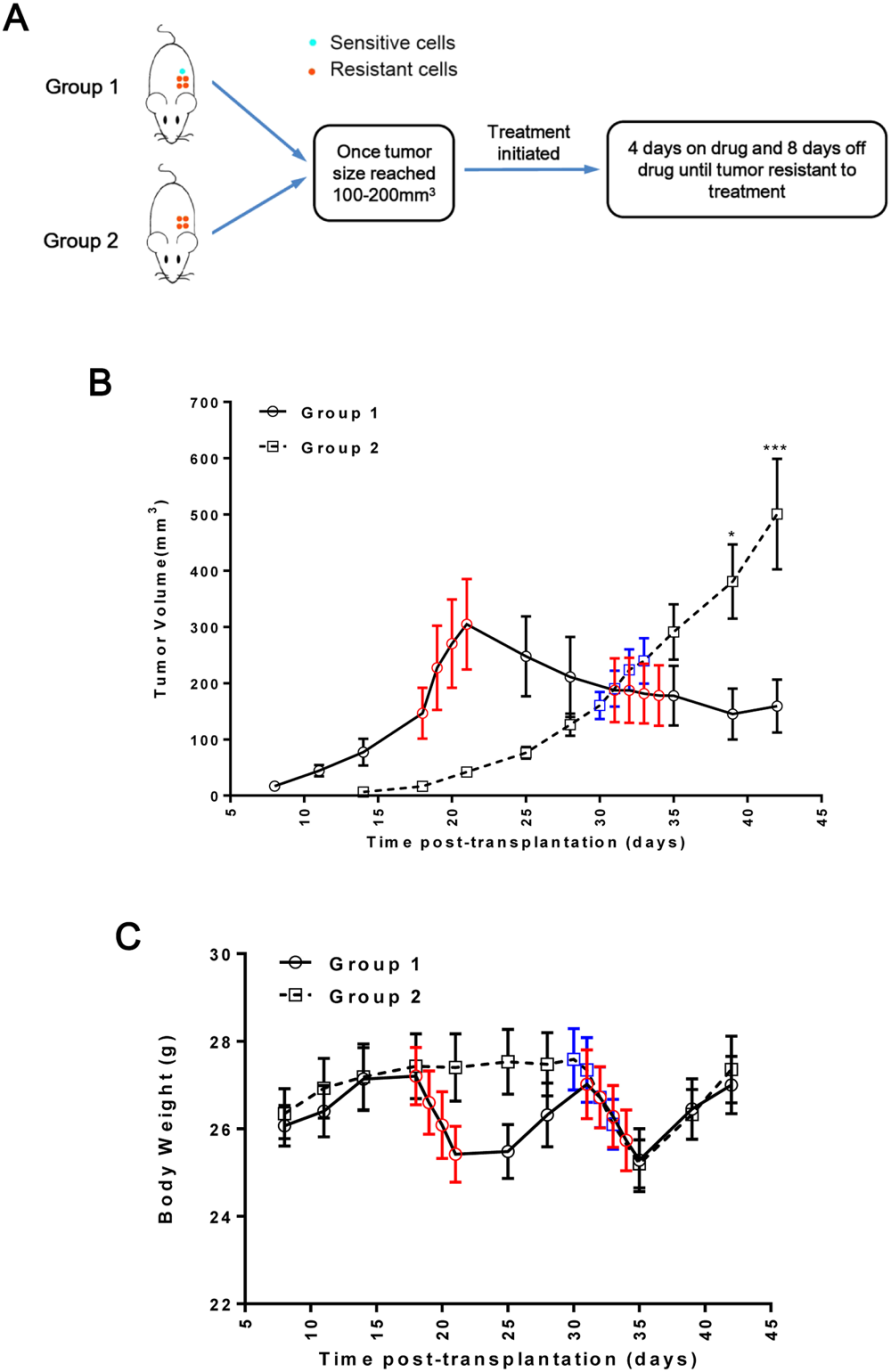
(**A**) Schematic representation of the tumor treatment assay in (**B**). For group 1, 2 × 10^6^ HeLa/ddp cells mixed with 5 × 10^5^ HeLa cells were injected subcutaneously into mice. For group 2, 2 × 10^6^ HeLa/ddp cells alone were injected subcutaneously into mice. (**B**) Tumor growth kinetics of group 1 (n = 6) and 2 (n = 7); both the red and blue dots represent one dose of ddp (2 mg/kg). The error bars indicate s.e.m. (**C**) Body weights of the mice in groups 1 and 2 throughout the treatment period; both red and blue dots represent one dose of ddp (2 mg/kg). The error bars indicate s.d.

We demonstrated that ddp-resistant cells were less fit than ddp-sensitive cells due to an enhanced antioxidant capacity. Hence, to further evaluate whether ddp-sensitive cells could delay the development of ddp-resistant cells during cisplatin treatment *in vivo*, two groups of tumors were established in Nu/Nu mice, and ddp treatment was initiated as shown in Fig. 5A. The tumors of group one (inoculated with 2 × 10^6^ HeLa/ddp cells mixed with 5 × 10^5^ HeLa cells) initially grew much faster than those of group two (inoculated with 2 × 10^6^ HeLa/ddp cells alone), but tumor size could still be controlled to 100–200 mm^3^ after 40 days by two cycles of treatment (Fig. 5B). However, while the tumors of group two grew slowly in the initial period, they did not respond to treatment, and their size easily grew beyond 500 mm^3^ after 40 days (Fig. 5B and Supplementary Fig. S4A). The body weights of the mice were decreased during treatment but could recover quickly after treatment (Fig. 5C). In conclusion, the growth of ddp-resistant cells could be controlled by maintaining a certain number of sensitive cells during treatment.

### Mathematical model of tumor growth and clinical treatment

To further evaluate the survival benefit obtained through different therapeutic strategies, we generalized a mathematical model to quantify the *in vivo* growth of a tumor with multiple subclones based on our experiments (Fig. 4C). As demonstrated by our experiments, tumor growth *in vivo* occurred in a power-law fashion, suggesting that tumor growth was strongly limited *in vivo*. Angiogenesis is widely considered to be the critical factor dominating *in vivo* tumor growth^18–21^. By modeling the effect of angiogenesis as the allometric growth of vessels, our theoretical model could quantitatively describe the power-law growth of the tumor. Thus, our model could clearly demonstrate that a limited angiogenesis rate could not always generate sufficient vessels to supply sufficient nutrients and oxygen for tumor cell proliferation as the tumors grew, which would lead to stronger competition between sensitive and resistant cells.

**Figure 6:**
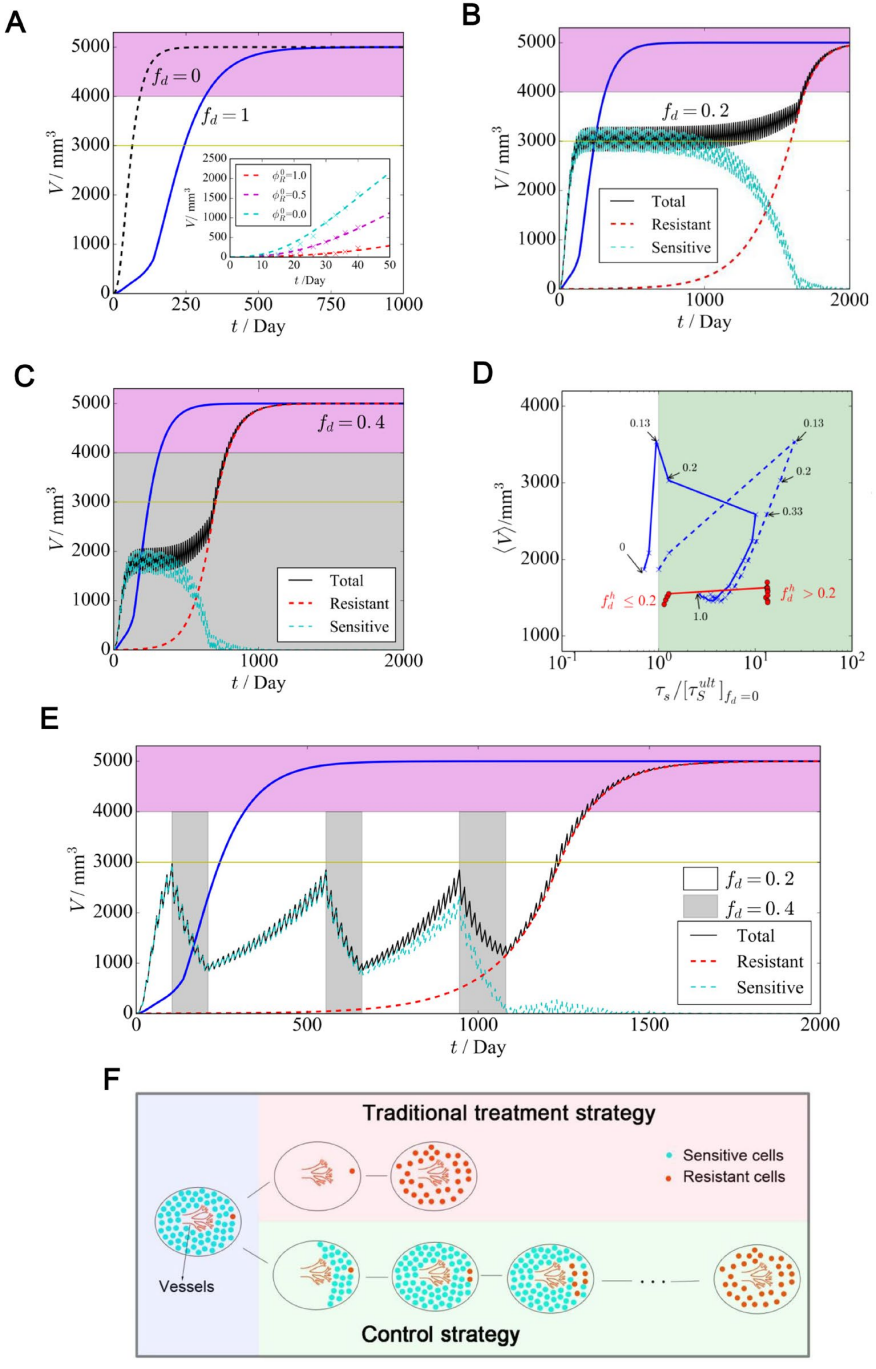
Model results of tumor growth and clinical treatment. (**A**) Tumor growth without dosing (*f*_*d*_ = 0) and with continuous high-dose therapy (*f*_*d*_ = 1). Inset: The mathematical model can model the tumor growth with different initial resistant cell fractions in drug-free cases. Here, the cross marks represented the growth of *in vivo* experiment (Fig. 4C) and the dashed lines represented the results of our mathematical model. (**B** and **C**) Tumor growth with different dosing frequencies. The blue line represents the traditional treatment strategy (*f*_*d*_ = 1). (**D**). For different fixed dosing frequencies, the scatter plot of the average tumor burden 〈*V*〉, ${\tau }_{{\rm{S}}}^{{\rm{ult}}}$ (blue dashed line) and ${{\rm{\tau }}}_{{\rm{S}}}^{\exp }$ (blue line) in comparison with the survival time of continuous dosing ${[{{\rm{\tau }}}_{{\rm{S}}}^{{\rm{ult}}}]}_{{f}_{d}=0}$ is shown. For adaptive therapy strategies with a varied dosing frequency, V and ${{\rm{\tau }}}_{{\rm{S}}}^{\exp }$ are marked with red dots. (**E**) Tumor growth under a varied dosing frequency. (**F**) Illustration of the traditional cure strategy and our control strategy.

More interestingly, our model predicted that tumor growth *in vivo* would show an irregular response to the dosing frequency *f*_*d*_. Here, *f*_*d*_ is defined as the fraction of time (in units of days) in which the growth of sensitive cells is suppressed during a course of treatment (15 days). We defined the ultimate survival time ${\tau }_{s}^{ult}$ as the time it took for the tumor to grow to a mortal level *V* ^*ult*^, and the expected survival time ${\tau }_{s}^{\exp }$ as the days during which tumor size could be controlled below a safe threshold *V* ^*exp*^ by therapy. As an example, based on the data from our *in vivo* experiments, we set the initial tumor volume as 1 mm^3^ (approximately *N*_0_ = 10^6^ cells), and the initial fraction of resistant cells as ${\varphi }_{R}^{0}=5 \%$ (The growth and control of tumors with other initial conditions are discussed in the Supplementary Materials). Additionally, we set *V* ^*ult*^ = 4000 mm^3^ and *V* ^*exp*^ = 3000 mm^3^. Under continuous dosing (*f*_*d*_ = 1), which corresponded to the traditional treatment strategy, there would be a 2-fold increase in overall survival compared with that in the dosing-free group (*f*_*d*_ = 0; Fig. 6A), but by killing all of the sensitive cells, the development of drug-resistant cells might be accelerated. In contrast, dosing at a certain frequency remarkably enhanced survival. As shown by our model, with a relatively low dosing frequency (*f*_*d*_ = 0.2; Fig. 6B), fewer sensitive cells were killed; thus, the growth of resistant cells could be strongly inhibited, and tumor size could be controlled for a longer time (approximately 1600 days), with ${\tau }_{s}^{ult}$ potentially presenting a 5-fold increase compared with continuous dosing. Such a result is consistent with our experimental results obtained *in vivo* (Fig. 5B). With a higher dosing frequency (*f*_*d*_ = 0.4; Fig. 6C), the patient’s tumor burden would initially be reduced (approximately 2000 mm^3^ for approsimately 600 days), but shortly thereafter, the tumor would rapidly grow to the mortal level *V* ^*ult*^. Under different initial conditions, compared with a continuous high dose, a low dosing frequency (0.1 ≤ *f*_*d*_ ≤ 0.2) could generally maximize ${\tau }_{s}^{ult}$, while a slightly higher dosing frequency (0.2 < *f*_*d*_ ≤ 0.4) would maximize ${\tau }_{s}^{\exp }$ by controlling the volume of the tumor at a “safe level” (Fig. 6D; detailed results are provided in the Supplementary Materials of Mathematical Modelling and Supplementary Fig. S5).

To design a therapy schedule with higher applicability, we first fully analyzed tumor growth under different dosing frequencies. The scatter plot (Fig. 6D) showed how different dosing frequencies could control the average tumor burden 〈*V*〉 and survival times (${\tau }_{s}^{ult}$ and ${\tau }_{s}^{\exp }$). Considering that controlling a large tumor in a patient will always represent a risk because this status is more likely to be perturbed by internal or external variations and might produce many side effects clinically, we must develop a practical way to both minimize the tumor burden and maximize survival time. According to previous analysis, a generalized control principle can be described as follows: when tumor size has grown to the upper boundary of the safe volume threshold, a high dosing frequency (e.g., *f*_*d*_ = 0.40) could reduce tumor size back to a lower level, whereas to maintain a higher fraction of sensitive cells, a lower dosing frequency (e.g., *f*_*d*_ = 0.20) should be applied. Therefore, we extend the idea of adaptive therapy^22^ by considering a compromise between a low and high dosing frequencies. As shown in Fig. 6E, by switching between the two dosing frequencies, both a long survival time (5-fold longer than under continuous dosing) and a relatively small tumor burden could be achieved.

## Discussion

Researchers are now beginning to realize the powerful ability of tumor cells to adapt to cytotoxic therapy accompanied by tumor heterogeneity, which has been confirmed in an increasing number of tumors^13, 23–26^. How to retard or even reverse this adaptability poses a significant challenge under these circumstances. Some studies have shown that a continuous dosing schedule will shift the evolutionary landscape in favor of drug-resistant clones^9^. Thus, drug-resistant clones may benefit from treatment and show rapid outgrowth^10^. The principle of adaptive therapy is to take advantage of the difference in fitness between drug-resistant and drug-sensitive cells and permit the fitter chemosensitive cells to grow so that they can, in turn, suppress the proliferation of less fit but chemoresistant cells^22^.

In order to investigate the fitness differences between ddp-resistant cells and ddp-sensitive cells, we developed three ddp-resistant cell lines through continuous and incremental exposure of the parental cells to various concentrations of ddp. We found that all three ddp-resistant cell lines showed slow growth rates, but we did not observed any other persistent phenotype changes such as morphology or organelle expansion in all three cell lines.

Based on our results, it was found that the ROS levels in ddp-resistant cells were significantly higher than in sensitive cells. Elevated ROS can increase the cytotoxicity of ddp by facilitating ddp-induced DNA damage or directly triggering MOMP^1^. The main cellular source of ROS is mitochondrial oxidative phosphorylation^15^, ROS are also produced in the endoplasmic reticulum and peroxisomes as well as during autoxidation process of small molecules^27^. There are multiple biological processes that participate in ddp resistance mechanisms^1^, and metabolic adaptation may also occur in ddp-resistant cells, since chemo-resistant cells have been reported to exhibit increased glycolytic metabolism in several studies^17, 28, 29^. These findings suggest that the production of ROS in ddp-resistant cells is probably a byproduct of resistance mechanisms or metabolic adaptation. There may certainly be additional factors that have not been addressed by the present study, and further studies are needed. Our results confirmed that acquisition of ddp resistance was accompanied by elevated ROS levels, which in turn decreased the proliferation of resistant cells. These observations could be explained by the fact that an elevation of ROS levels can induce slow proliferation, while the detoxification of elevated ROS levels results in an excess substrate cost.

The significant difference between the growth rate of ddp-sensitive cells and that of ddp-resistant cells is a fundamental factor in our model. The elevated ROS level in ddp-resistant cells plays a key role in the slower growth rate of ddp-resistant cells, which was confirmed by our *in vitro* experiment. In our model, the growth of ddp-resistant cells would be remarkably accelerated if the adaptive defect of ddp-resistant cells resulting from ROS accumulation were ignored. Although ROS are not an explicit parameter in our model, the slower growth rate of ddp-resistant cells *in vivo*, as indicated by our mathematical model, was influenced by ROS accumulation, indicating that our mathematical model already considered effects related to ROS accumulation.

Glucose is an important substrate for tumor growth, and the energetic cost of resistance becomes a significant burden for doxorubicin-resistant cells under glucose restricted conditions^17^. In our study, ddp-resistant cells had already exhibited significantly slower growth rates under glucose unrestricted conditions (2 g/L), so we did not further investigate the effects of substrate competition under glucose restricted conditions, which would be interesting to pursue in future studies.

Angiogenesis is an essential factor in tumor growth^30^ and represents the pivotal limitation to tumor expansion. Our experiments suggested that angiogenesis also played an important role in the selection of ddp resistance. For example, as the proliferation of resistant cells requires more nutrition, when the diffusion rate was kept constant, the average number of vessels became a restrictive condition for the growth of resistant cells *in vivo* compared with the parental cells. All of the results obtained both *in vitro* and *in vivo* indicated that resistant cells possessed an extremely poor expansion ability in environments lacking oxygen and nutrition but exhibited rapid outgrowth in the presence of abundant of nutrients and oxygen. The vascular network that had formed to supply tumor cells with the necessary nutrients for their continued growth did not disappear after tumor regression during treatment. Consequently, drug-resistant cells that survived cytotoxic therapy might have benefited from these vessels and had a sufficient nutrient supply and space to regrow rapidly without competitors. Our results demonstrated that maintaining a certain number of sensitive cells to compete with resistant cells could retard the development of ddp resistance (Fig. 6E and F). This is also a possible explanation for the equal or better benefit in terms of overall survival associated with intermittent chemotherapy compared with continuous chemotherapy using platinum-based drugs observed in some clinical trials^31, 32^.

Due to experimental limitations related to the toxic and side-effects of ddp, we built a mathematical model of tumor growth and clinical treatment parameterized based on *in vivo* studies, to further quantify the survival benefit of different dosing strategies. In previous studies^17, 22^, inhibition rates have usually been considered to be dependent on the average or local fraction of other subclones. However, such simple assumptions cannot fully describe the essential aspects of tumor growth *in vivo* because a high proliferation rate and a high angiogenesis rate might be coupled together. Our allometric growth model with dynamic inhibition rates effectively took the effects of angiogenesis into account, and our predictions regarding tumor growth *in vivo* may therefore be more consistent with actual conditions.

Our model implied that by taking advantage of the competition between different subclones, a periodic dosing schedule with an appropriate drug-free gap time could lead to a longer survival time than continuous high-dose therapy. Moreover, our model could predict the survival times of a patient under different chemotherapeutic strategies with different initial conditions. Similar to Gatenby’s adaptive therapy^22^, our method was also able to control the tumor to a certain size, and patient survival could be greatly extended compared with that associated with a traditional treatment strategy. More interestingly, by switching between two dosing frequencies according to the tumor volume (Fig. 6E), it was possible to control both the growth of drug-resistant cells and tumor size. This model may provide a platform for designing a more effective treatment strategy with platinum-based drugs and a framework for designing individualized dosing schedules.

Our model shares the same fundamental principles as preexisting metronomic therapy and adaptive therapy strategies^22, 33^, and it is generally expected that patient survival will be prolonged by adjusting the treatment interval or dosage. However, there are still some important differences between our model and other preexisting models. To describe tumor growth *in vivo* with a higher accuracy, the parameters in our mathematical model are fully based on *in vivo* experiments. Our model can precisely model the nonexponential growth of the tumor *in vivo*. We effectively considered angiogenesis accompanied by tumor growth in our model, so that the competition between subclones could be better quantified. Our therapeutic protocol, which controlled tumor growth by switching between two dosing frequencies, showed some advantages in the control of tumor growth. For example, compared with fixed-frequency metronomic therapy, our strategy is relatively flexible in addressing different cases. Conversely, compared with adaptive therapy, our strategy is more regular, and it is therefore not necessary to constantly change the dosing timetable.

There was study suggested that complete withdrawal of drug between treatments might cause rapid rebound of tumor growth^34^, which seemed inconsistent with our model. Both the growth kinetics of tumors and chemotherapy drugs were different in these two studies, and most importantly, this was related to time length between treatments. If we prolonged treatment interval time, then rapid rebound of tumor growth would also be observed in our experiment.

Our results provide direct experimental evidence that ddp-resistant cells are less fit than their parental cells in the absence of the drug. The progression of ddp-resistant cells will be accelerated if we kill all of the sensitive cells during treatment. In contrast, if we maintain a certain number of sensitive cells to compete for nutrient and space with resistant cells, we can significantly suppress the growth of resistant cells and prolong patient overall survival time. We have two options to either choose resistant or sensitive cells to grow based on the tumor evolution dynamics. Ddp-resistant cells grow slowly but in a manner that is beyond our control, while sensitive cells exhibit a rapid growth rate but respond to drug treatment. We may be not able to overcome the development of ddp resistance at present, but the maintenance of ddp-sensitive cells may help us to control its progression. Our modeling indicates that a significant survival benefit may be achieved through appropriate treatment intervals, which may also have the potential to reduce the side-effects and cost of drugs.

## Materials and Methods

Our study was approved by the Ethics Review Committee for Animal Experimentation at Drum Tower Hospital (Nanjing, China). All animal procedures were performed in compliance with guidelines set by the Animal Care Committee, and all efforts were made to reduce the possible pain and discomfort of the animals.

### Cell Culture

HeLa, HGC27, and AGS cells were purchased from the Type Culture Collection of the Chinese Academy of Sciences, Shanghai, China. HeLa/ddp, HGC27/ddp, and AGS/ddp cell lines were developed through continuous and incremental exposure of the parental cells to various concentrations of ddp (P4394, Sigma) (Supplementary Fig. S1A and B). HeLa-RFP cells were derived via lentiviral transduction of HeLa cells with pGLVU6/RFP (GenePharma). All cell lines were cultured in RPMI 1640 (Invitrogen) supplemented with 10% FBS (Biological Industries, BI) and 1% penicillin-streptomycin (50 µnits/ml, 50 µg/ml, Invitrogen) in a 5% CO_2_ environment. All sensitive-cell lines and resistant-cell lines were authenticated via short tandem repeat profiling analysis in 2014. All ddp-resistant cell lines were identical to the parental cell lines, respectively.

### Establishment of ddp-resistant cell lines

The initial dose of ddp was 1/10 IC_50_ (70 ng/ml, 120 ng/ml, and 200 ng/ml in HeLa, HGC27 and AGS cells, respectively), and the dose was increased by adding 70–200 ng/ml to each previous dose. The next dose was administered until the cells entered a stable growth period. The dose responses of these 6 cell lines were assessed for ddp, and the IC_50_ values were determined to be 0.75 ± 0.12 μg/ml (HeLa), 7.50 ± 1.70 μg/ml (HeLa/ddp), 1.21 ± 0.10 μg/ml (HGC27), 9.69 ± 0.72 μg/ml (HGC27/ddp), 2.32 ± 0.20 μg/ml (AGS) and 15.14 ± 1.56 μg/ml (AGS/ddp) (Supplementary Fig. S1). HeLa/ddp, HGC27/ddp, and AGS/ddp cells were maintained in the presence of 0.7, 1.2, or 2.0 μg/mL ddp, respectively, until 1 week before the experiments to ensure maintenance of the resistant phenotype.

### Cell growth analysis

Cells were plated in 24-well plates at 1 × 10^5^ cells per well in 0.5 ml of media (5 × 10^4^ cells per well for HGC27 and AGS). The medium was changed daily. At the indicated time points, cells in triplicate wells were trypsinized and counted with a Hand-held Automated Cell Counter (Scepter 2.0, Millipore).

### CFSE assay

Cells were labeled with 5-(and 6)-carboxyfluorescein diacetate succinimidyl ester (CFSE) (eBioscience) according the manufacturer’s instructions. The labeled cells were cultured or treated as desired and then analyzed by flow cytometry (BD Bioscience) at the end of the experiment. Proliferation was calculated as follows. *C*_0_, *C*_1_ and *C*_2_ represented the initial cell numbers, control group cell numbers and experimental group cell numbers, respectively. *n*_1_ and *n*_2_ represented the average population doublings of the control and experimental groups, respectively. *FI*_0_, *FI*_1_ and *FI*_2_ represented the initial average fluorescence intensity and the average fluorescence intensity of the control and experimental groups, respectively.

$${\rm{Relative}}\,{\rm{proliferation}}=\frac{{C}_{2}}{{C}_{1}}=\frac{{C}_{0}{2}^{{n}_{2}}\,}{{C}_{0}{2}^{{n}_{1}}\,}=\frac{{C}_{0}F{I}_{0}/F{I}_{2}\,}{{C}_{0}\,F{I}_{0}/F{I}_{1}}=\frac{F{I}_{1}}{F{I}_{2}}$$

### Colony-formation assay

Cells (500–1000) were plated in 6-well plates. The medium was not altered throughout the course of the experiment. After 10–14 days, colonies were fixed in methanol and stained with 0.5% crystal violet.

### ROS quantification

Cells were incubated with 5 μM 2′,7′-dichlorofluorescin diacetate (DCFDA, Sigma) for 25 min. Excess DCFDA was removed, after which the cells were washed twice with PBS, and labeled cells were then trypsinized and resuspended in PBS. The mean fluorescence intensity was analyzed by flow cytometry.

### EdU assay

Cells were labeled with the Click-iT® Plus EdU Imaging Kit (Invitrogen) according the manufacturer’s instructions. The percentage of EdU^+^ cells was estimated by counting an average of 500–1000 cells per field from 3 randomly selected sample regions using ImageJ software.

### Analysis of apoptosis and the cell cycle distribution

*In vitro* analyses of apoptosis rates and the cell cycle were performed using the Annexin V-FITC apoptosis detection kit (Miltenyi Biotec) and the Cycletest^TM^ Plus DNA Reagent Kit (BD Bioscience), respectively, according the manufacturers’ instructions.

### Western blotting analysis

Protein lysates were electrophoretically separated by SDS-PAGE, then transferred to polyvinylidene fluoride membranes and immunoblotted with the desired antibodies. The blots were developed with ECL Western blotting reagents (Millipore). The following antibodies were used: Cleaved Caspase-3 (9661, CST), Caspase-3 (9662s, CST) and β-Actin (A5441, Sigma).

### Xenograft experiments

Male Nu/Nu mice were purchased from Vital River Laboratories. For subcutaneous xenografts, 1 × 10^6^ cells were suspended in 0.1 ml of 50% Matrigel (BD Biosciences) solution in RPMI 1640 and injected subcutaneously into the lower flank of 4-week old Nu/Nu mice. Tumor volumes were monitored based on bi-weekly measurements of tumor diameters conducted using electronic calipers. Once the tumor volume reached 1,000–2,000 mm^3^, the animal was euthanized, and the tumor was harvested for further experiments. Tumor volumes were calculated by the following formula: 1/2 × length × width^2^.

For ddp treatment, once tumor size reached 100–200 mm^3^, we subcutaneously injected 2 mg/kg ddp for four consecutive days and then withdrew treatment for eight days before another cycle was initiated, until tumor became resistant to treatment.

### Immunohistochemistry

For histological analyses, tumor xenografts were fixed in 10% buffered formalin (Sigma) and embedded in paraffin. Paraffin sections were then processed for either hematoxylin and eosin (H&E) staining or immunohistochemistry. The antibodies used for immunostaining included Ki67 (ab8191, Abcam) and CD31 (ab28364, Abcam). Scoring of the expression of each marker was performed as follows: the percentages of Ki67^+^ and CD31^+^ cells were estimated by counting an average of 1,500–2,000 cells per sample from 3 randomly selected regions of the xenografts using ImageJ software. Vessel density was scored by counting the number of CD31 vessels per field from 4-6 randomly selected fields in the tumor, and the average was calculated.

### Terminal deoxynucleotidyl transferase dUTP nick-end labeling (TUNEL) assay and RFP ratio analyses

TUNEL assays were carried out using the In Situ Cell Death Detection Kit (Roche) according to the manufacturer’s instructions. For the analyses of RFP ratio, tumor xenografts were frozen in liquid nitrogen, after which 5-μm sections of the frozen xenografts were stained with DAPI according to standard protocols, and images were acquired with a fluorescence microscope (ZEISS). The percentages of TUNEL^+^ and RFP^+^ cells were estimated by counting an average of 1500–2000 cells per sample from 3 randomly selected tumor regions using ImageJ software.

### Allometric growth

We applied allometric growth, as first introduced by West *et al.* ^18^, West and Brown^19^, and Guiot *et al.* ^20^ to model the *in vivo* growth of tumors. A general allometric scaling law with a 3/4 exponent could be derived from the fractal structure of the capillary distribution and fit many physiological processes well, including tumor growth^19, 21^. Thus, by effectively taking into account angiogenesis, allometric growth could not only elucidate the sigmoidal growth of tumors well^35^, but also showed a good fit to our *in vivo* experimental data. In our model, allometric tumor growth was generally described by the differential equation $\frac{dN}{dt}=a{N}^{3/4}-bN$. For resistant cells (R) and sensitive cells (S), there were different proliferation rates *a*_*R*_, *a*_*S*_ and death rates *b*_*R*_, *b*_*S*_, respectively.

### Dynamic inhibition rate

To precisely model the *in vivo* growth of tumors, the competition among different subclones regarding the occupation of vessels should be correctly evaluated. Due to the different angiogenesis rates of different subclones, the inhibition rate depended not only on the temporary fraction of subclones, but also on the history of tumor and vessel growth (i.e., both the fractions of subclones and the effective proliferation rate of subclones (${\hat{a}}_{R},{\hat{a}}_{S}$) were dynamically changing). In our model, dynamic inhibition rates were parameterized based on *in vivo* experiments. Details are provided in Supplementary Materials of Mathematical Modelling.

### Statistical analyses

Unless otherwise indicated, all experiments were performed in triplicate, with the mean and standard deviation (s.d.) being reported where appropriate. Differences between treatments were evaluated using ANOVA or Student’s t test. The Chi-square test was applied to compare differences in proportions (Tumor formation rate). Differences were considered significant at P < 0.05 (*P < 0.05; **P < 0.01; and ***P < 0.001).

## Electronic supplementary material

Supplementary information
