# Supplementary material for: A Strategy to Delay the Development of Cisplatin Resistance by Maintaining a Certain Amount of Cisplatin-Sensitive Cells
Source: Sci Rep. 2017 Mar 27;7:432. doi: 10.1038/s41598-017-00422-2 (PMC5428423; doi:10.1038/s41598-017-00422-2)
Supplement: Supplementary file 1 — Supplementary information [file 41598_2017_422_MOESM1_ESM.pdf]

Title: A Strategy to Delay the Development of Cisplatin Resistance by Maintaining a Certain Amount of Cisplatin-Sensitive Cells

Guihua Duan, Qianyuan Tang, Hongli Yan, Lijuan Xie, Yun Wang, Xi Emily Zheng, Yuzheng Zhuge, Shanshan Shen, Bin Zhang, Xiaoqi Zhang, Jun Wang, Wei Wang and Xiaoping Zou

Supplementary figure legends

Figure S1. A. An illustration of establishment of three ddp-resistant cell lines. **B.** The IC_{50} values for HeLa, HeLa/ddp, HGC27, HGC27/ddp, AGS and AGS/ddp cells.

Figure S2. A. Representative wells of the clonogenic growth experiment depicted in Figure 1B. **B.** Relative clonogenic growth of HGC27, HGC27/ddp, AGS and AGS/ddp cells under conditions indicated. GSH (1 mM) was added to the medium the following day.

Figure S3. A. Representative images of indicated staining in Figure 4D. Scale bars, 100 μm. **B.** Growth of HeLa and HeLa-RFP cells, the medium was exchanged with normal medium every day.

Figure S4. A. Therapeutic activity of cisplatin in xenograft tumor mice shown in Figure 5B.

Figure S5. The contour plot of the folds that the periodic dosing could increase the patients' ultimate or expected survival time compared with continuous dosing. **A** and **B.** With different initial fraction of resistant cells (left: $\phi_R^0 = 0.2$, middle: $\phi_R^0 = 0.5$, and right: $\phi_R^0 = 0.8$), for different initial volume of the tumor V_0, by changing the dosing frequency f_d, the contour plot of ultimate survival time increase ratio (a), and expected survival time increase ratio (b). **C** and **D.** With different initial volume of the tumors (left: $V_0 = 1\ \mathrm{mm}^3$, middle: $V_0 = 10\ \mathrm{mm}^3$, and right: $V_0 = 100\ \mathrm{mm}^3$) for different initial fraction of resistant cells ϕ_R^0, by changing the dosing frequency f_d, the contour plot of ultimate survival time increase ratio(c), and expected survival time increase ratio(d).

Supplemental Figure 1

A

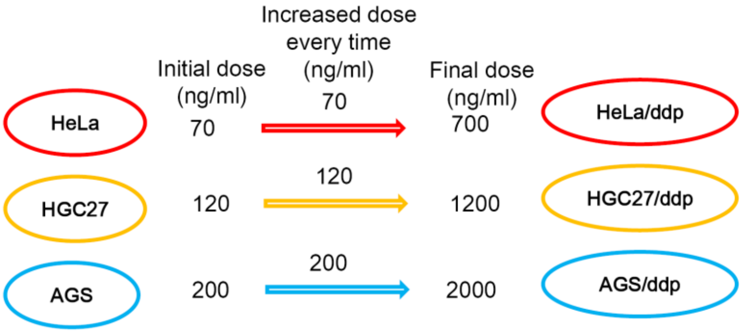

B

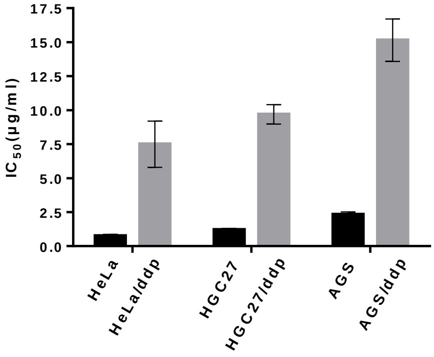

Supplemental Figure 2

A

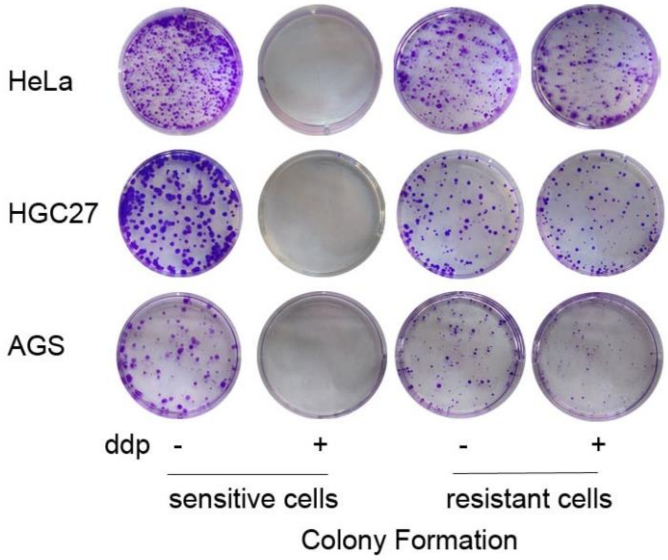

B

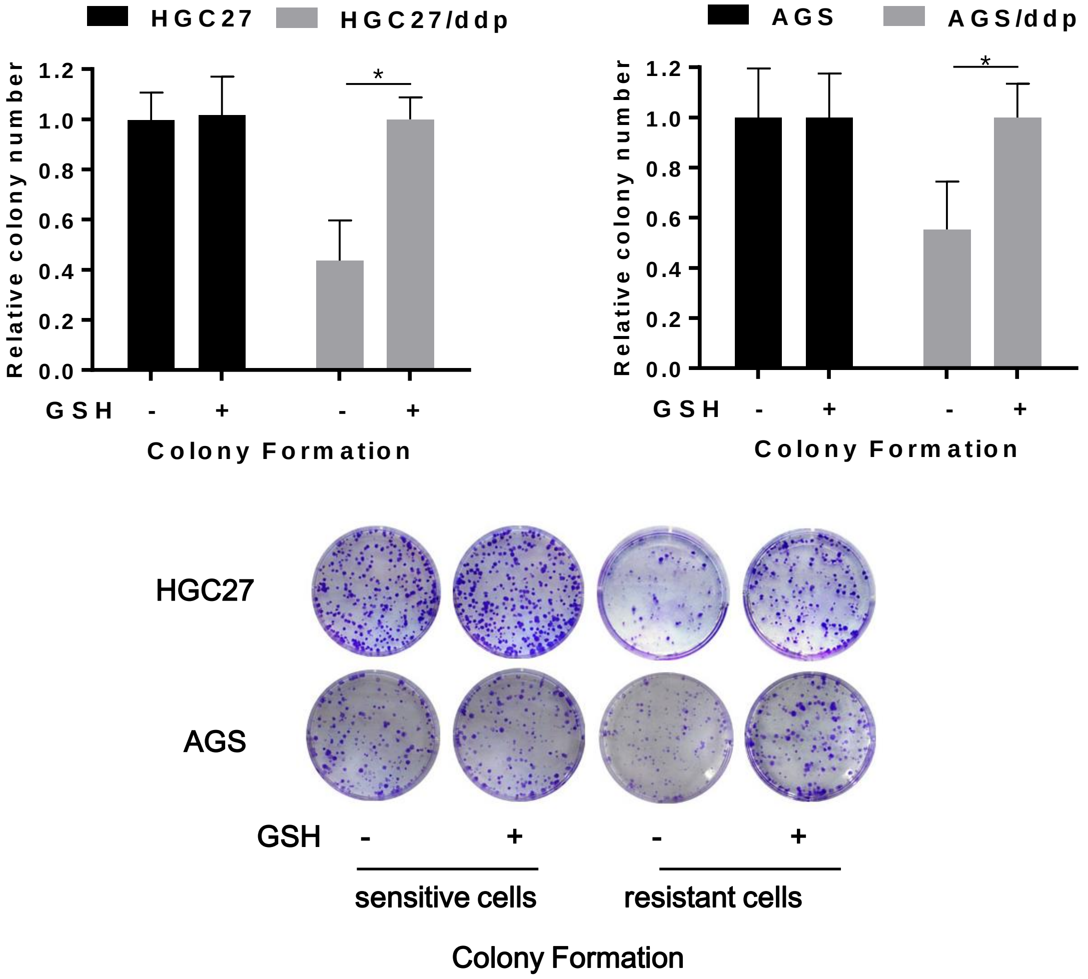

Supplemental Figure 3

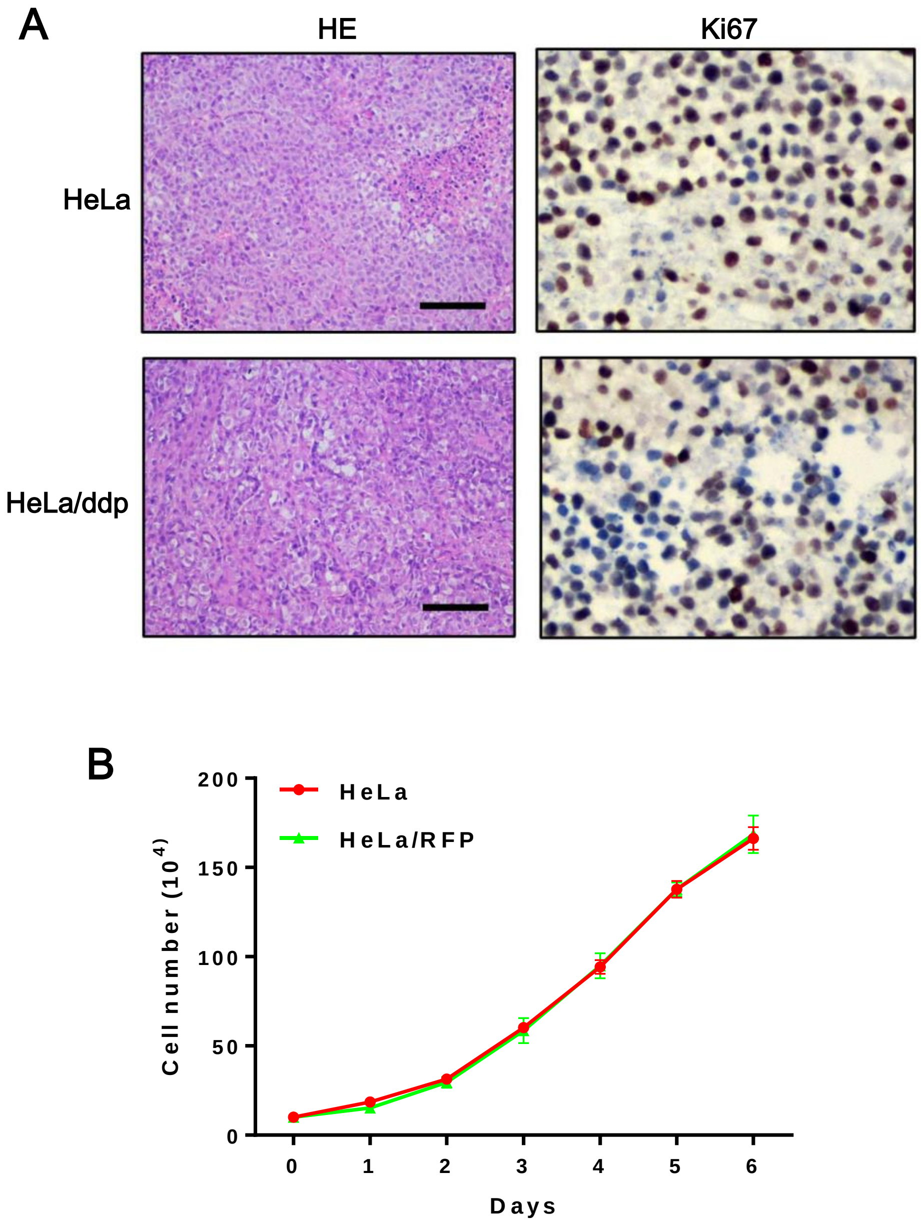

Supplemental Figure 4

A

Group 1

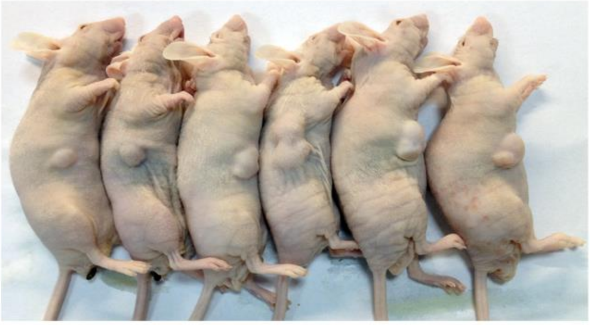

Group 2

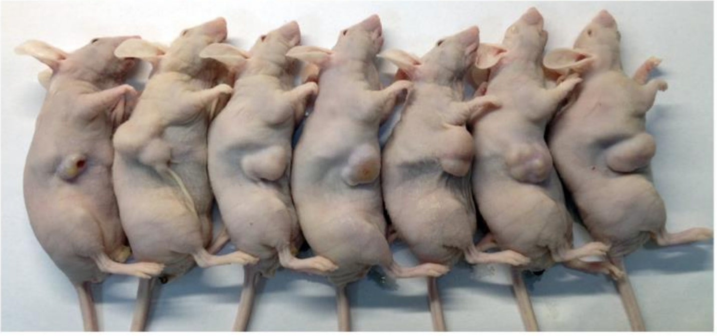

Supplemental Figure 5

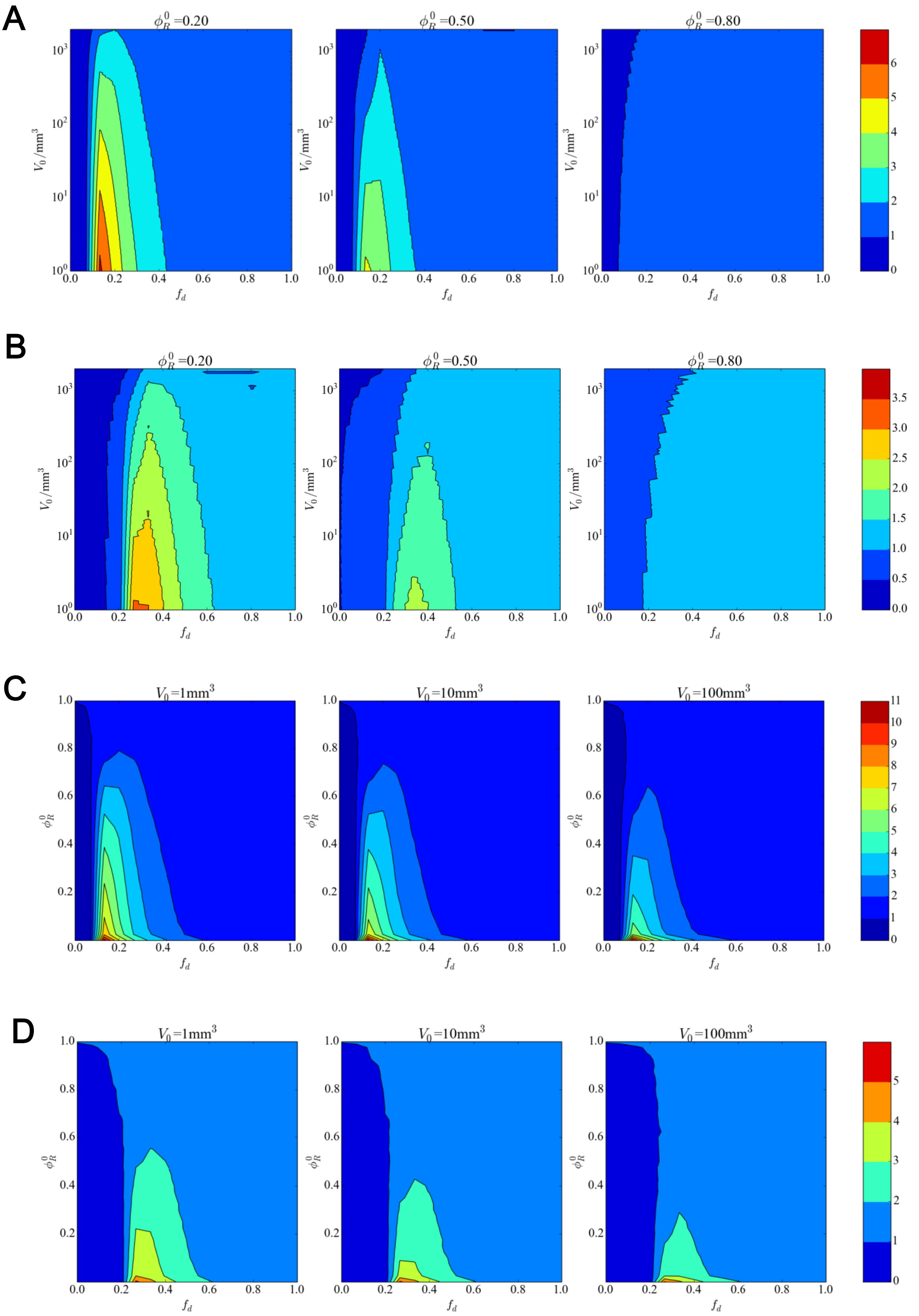

Supplementary Materials of Mathematical Modeling

"A Strategy to Delay the Development of Cisplatin Resistance by Maintaining a Certain Amount of Cisplatin-Sensitive Cells"

Guihua Duan, Qianyuan Tang, Hongli Yan, Lijuan Xie, Yun Wang, Xi Emily Zheng, Yuzheng Zhuge, Shanshan Shen, Bin Zhang, Xiaoqi Zhang, Jun Wang, Wei Wang and Xiaoping Zou

As discussed in the main text, based on *in vivo* experiments, we generalized a mathematical model that implicitly considered the angiogenesis coupled with the multi-subclone tumor growth, and we applied such a model to evaluate the therapeutic strategy for the tumors with different initial conditions. In this Supplementary Text, the details of our mathematical model to quantify the tumor growth and the clinical treatment were described; and in the last page, all the equations and the values of the parameters were listed.

1 Model details

1.1 The growth of a single subclone

There were already many models describe tumor growth following a sigmoidal curve. Generally, all the sigmoidal tumor growth could be described by the equation [1]:

$$\frac{dN}{dt} = aN^{\alpha} - bN^{\beta}.$$

Note that there were two parameters (α and β) in the equation, the determination of the parameter was an important question in modeling the tumor growth. Strikingly, when the incoming oxygen and nutrients were transported through hierarchical branching network systems, then the allometric growth law could successfully describe the ontogenetic development of different organisms [2, 3]. In fact, the allometric law could be recognized as a general model [2]. Therefore, the tumor growth, which supported by the hierarchical branching vessel network, could also be described by the allometric growth [4, 5]. Considering that all the tumor cells might die, but only those cells near the vessels (oxygen and nutrients) were capable of division, the cell proliferation could only happen at a lower fractal dimension than the cell death occurred, i.e., $\alpha < \beta = 1$. And it could be obtained that $\alpha = 3/4$ according to the fractal-like structure of the energy distribution [5]. So, for a tumor consists of a single subclone, e.g., resistant cells (R), then the *in vivo* growth of tumor cells could be described by the differential equation:

$$\frac{dN_R}{dt} = a_R N_R^{\frac{3}{4}} - b_R N_R,$$

in which a_R and b_R were the proliferation rate and the death rate of the resistant cells, respectively. In this situation, the amount of the vessels (per cell) for such a tumor could be estimated as proportional to $N_R^{-1/4}$, i.e., as the tumor grew, the amount of vessels would gradually become a restriction.

Such a growth equation indicated that the number of the resistant cells in the steady state ($\frac{dN_R}{dt} = 0$) was: $N_R^* = \left(\frac{a_R}{b_R}\right)^4$, where N_R^* was the asymptotic cell numbers that

the tumor could grow up to. For a given N_R^*, the death rate of the resistant cells $b_S = a_S {N_R^*}^{-\frac{1}{4}}$.

Similarly, for the growth of the sensitive cells (S):

$$\frac{dN_S}{dt} = a_S N_S^{\frac{3}{4}} - b_S N_S,$$

where a_S and b_S were the proliferation rate and the death rate of the sensitive cells, respectively. The steady state of the sensitive cells $N_S^* = \left(\frac{a_S}{b_S}\right)^4$, and the death rate of the sensitive cells $b_S = a_S {N_S^*}^{-\frac{1}{4}}$. As observed in our experiments, the growth of resistant cells was substantially slower than that of sensitive cells in the drug-free situation. This condition was quantitatively considered in our model. According to our *in vivo* experiment (as shown in Fig. 4C), it was estimated that $a_R/a_S \approx 0.35$.

In our model, the cell numbers of each subclone could be obtained by solving the differential equations numerically. In this work, the fourth-order Runge-Kutta method was employed.

1.2 The growth of multiple subclones without drugs

Considering a tumor consists of two subclones (resistant and sensitive cells), the total cell number $N = N_R + N_S$. And according to our *in vivo* experiments, the growth of sensitive cells could be highly suppressed by the resistant cells, which indicated the competition between the two subclones. Still, the allometric growth would be a general restriction, so we could first write the growth equation of the total cell numbers as:

$$\frac{dN}{dt} = \hat{a} N^{\frac{3}{4}} - \hat{b} N.$$

Due to the competition, the growth rate of the tumor was different from the independent growth of each subclone ($\hat{a} \neq a_S$ and $\hat{a} \neq a_R$). Parameter $\hat{a}$ indicated the competition between different subclones, and as the *in vivo* suggested, $a_R \leq \hat{a} \leq a_S$.

In our model, for simplification, we assumed that the asymptotic cell numbers did not rely on the fraction of each subclone. For the tumors *in vivo*, no matter how the fractions of each subclones diverged, the tumor cells could always grow to the same limit: $N_S^* = N_R^* = N^*$. Note that $\hat{a}$ and $\hat{b}$ were the parameters describing the growth of the tumor with multiple subclones. We defined the fraction of sensitive cells $\phi_S = N_S/N$ and that of resistant cells $\phi_R = N_R/N = 1 - \phi_S$.

With the same initial number of cancer cells and the same environments *in vivo*, the value of $\hat{a}$ would be a function of the initial fraction of subclones. Note that $\hat{a}_R \neq a_R$ and $\hat{a}_S \neq a_S$. According to our *in vivo* experiment, we took the growth rate of a tumor

(consists of sensitive cells and resistant cells) as linear interpolation of the independent growth rate of two subclones. For example, when the fraction of sensitive cells was ϕ_S, then the growth rate $\hat{a}$ was a function of ϕ_S and $\hat{a}(\phi_S) = a_R(1 - \phi_S) + a_S\phi_S$.

1.2.1 The asymptotic volume of the tumor.

It was assumed that the asymptotic volume of the tumor (consists of a single subclone) was always a constant, that was, $N^* = (a_R/b_R)^4 = (a_S/b_S)^4$. For the tumor growth in mice, we set the asymptotic volume $V^* = 5000$ mm^3 (approximately 20% of the average body mass of a mouse, and $V^* \sim N^*$), which was in the same order as determined by experiments [7].

When there was a strong limitation of the resources (e.g., taking the diffusion of oxygen or nutrients into account), the inhibition rates between subclones would increase. In these situations, with lower $\hat{a}_S$ and $\hat{a}_R$, the steady state volume (i.e., asymptotic cell numbers) of tumor with multiple subclones would be smaller. Our model could be easily generalized to such cases. In such cases, a lower fraction of resistant cells could be maintained with a smaller tumor volume [6], so the tumor growth would be much easier to control.

1.2.2 Weak inhibition of resistant cells.

In our model, for simplicity, we ignored the changes of $\hat{a}_R$ and took $\hat{a}_R \approx a_R$ and $\hat{b}_R \approx b_R$. This was because a_R was much smaller than a_S (as suggested by *in vitro* and *in vivo* experiments).

1.2.3 Dynamic inhibition rate.

To precisely model the *in vivo* growth of tumor, we introduced the dynamic inhibition rate to model the competition between the subclones based on our experiments. As discussed in the main text, the dynamic inhibition rate was a consequential effect of the angiogenesis. On the one hand, the two subclones would directly compete for the occupation of vessels. On the other hand, as an additional effect, since the vessel growth rate varied for different subclones, the potential ability for angiogenesis would also highly depend on the fractions of subclones. By taking allometric growth coupled with competitions between subclones, our model could take both of effects into account. In this way, our predictions for the tumor growth *in vivo* could be more consistent to the actual conditions. Here, we listed the model details of how we introduced the dynamic inhibition rate into our model.

(1) Different subclones compete for the occupation of vessels.

Due to the competition among subclones, both the fractions and the growth rates of subclones ($\hat{a}_R$ and $\hat{a}_S$) were dynamically changing. For tumors with multiple subclones, we first could write the growth equations of each subclone respectively:

$$\frac{dN_R}{dt} = \hat{a}_R N^{-\frac{1}{4}} N_R - \hat{b}_R N_R,$$

$$\frac{dN_S}{dt} = \hat{a}_S N^{-\frac{1}{4}} N_S - \hat{b}_S N_S.$$

The above equations shared the same format. And they could be understood as the growth of cancer cells coupled with angiogenesis: the average amount of vessels per cell were proportional to $N^{-1/4}$, and parameters $\hat{a}_R$ and $\hat{a}_S$ had taken both the allometric growth and the competition in growth into account.

Here, the above equations showed that how resistant cells and the sensitive cells were competing for the "occupation of vessels". As the tumors grew, the total amount of vessels (and the proliferation rate of cancer cells) would be approximately proportional to $N^{3/4}$, while the death rate would approximately proportional to N. For every tumor cell, the average amount of vessels per cell were proportional to $N^{-1/4}$, which would decrease as N increase, so our equations could describe the phenomenon that the tumor growth rate would finally decrease. During such a process, the total amount of vessels that resistant cells (N_R) had occupied could be estimated as $N^{-1/4}N_R$; similarly, for every sensitive cell, the total amount of vessels that sensitive cells (N_S) had occupied could be estimated as $N^{-1/4}N_S$. According to the equations above, the proliferation rates of subclones were proportional to the term of $N^{-1/4}N_R$ and $N^{-1/4}N_S$, respectively, which reflected the competition between the two subclones. In a mean field scenario, the subclone with a lower fraction would only occupy a smaller part of the vessels, and its growth would be inhibited.

Summing up the two equations above, one could obtain that:

$$\frac{dN}{dt} = N^{-\frac{1}{4}} \left(\hat{a}_R N_R + \hat{a}_S N_S\right) - \left(\hat{b}_R N_R + \hat{b}_S N_S\right).$$

We could compare the equation above with the equation $\frac{dN}{dt} = \hat{a}N^{\frac{3}{4}} - \hat{b}N$. A remarkable point of the equations above was that the growth of the total tumor had to be consistent with the growth of multiple subclones with competition. For the *in vivo* growth, the total tumor growth should equal to the growth of the two subclones, so we could derive that:

$$\hat{a}_R\phi_R + \hat{a}_S\phi_S \approx \hat{a} \approx a_R\phi_R^0 + a_S\phi_S^0,$$

$$\hat{b}_R\phi_R + \hat{b}_S\phi_S \approx \hat{b} \approx b_R\phi_R^0 + b_S\phi_S^0.$$

Here, ϕ_S^0 and ϕ_R^0 were the initial fractions of sensitive cells and resistance cells, respectively.

(2) Potential ability of angiogenesis as a restriction of the tumor growth.

According to the two equations above, we could obtain:

$$\hat{a}_S \approx \frac{a_R\phi_R^0 + a_S\phi_S^0 - \hat{a}_R\phi_R}{\phi_S} \approx \frac{a_S\phi_S^0}{\phi_S}\left(1 - \frac{a_R}{a_S}\right) + a_R,$$

$$\hat{b}_S \approx \frac{b_R\phi_R^0 + b_S\phi_S^0 - \hat{b}_R\phi_R}{\phi_S} \approx \frac{b_S\phi_S^0}{\phi_S}\left(1 - \frac{a_R}{a_S}\right) + b_R.$$

For a tumor consists of multiple subclones, considering the competitions between subclones, each subclone would grow much slower than their independent growth ($\hat{a}_S < a_S$). As mentioned in the "Method" part, the growth of the tumor was restricted by the rate of angiogenesis. From the equations above, it could be concluded that the inhibition rate of a subclone would dynamically change depending on the ability of angiogenesis, which was related to the change of cell fraction with respect to the initial states (ϕ_S^0/ϕ_S).

1.3 The growth of multiple subclones with drugs

With drug dosed, the growth of sensitive cells was suppressed, so a lower $\hat{a}_S$ should be applied. For simplification, we still took $\hat{a}_R \approx a_R$ and $\hat{b}_R \approx b_R$. And the similar allometric growth could still remain.

1.3.1 The total amount of vessels is not reduced after treatment.

In the drug-free cases, we considered the average amount of vessels (per cell) to be proportional to $N^{-1/4}$. However, the size of the tumor (the total cell number) might decrease after dosing, but the vessels would not be reduced. Therefore, when the tumor size was reduced, the total amount of the vessels could be estimated as: $N_m^{-1/4}(t) \cdot N_m(t)$, where $N_m(t)$ was the maximum number of total cells during the growth history from the initial time to time t. When the number of cancer cells were reduced to N, the amount of vessels per cell could be estimated as $N^{-1}N_m^{3/4}(t)$.

1.3.2 Dosing frequency

In our model, we defined the dosing frequency f_d as the fraction of time (in unit of days) with drug dosing in a course of treatment (15 days). During this time, the growth of the sensitive cells were suppressed. In order to fully reveal the effect of the competition between subclones *in vivo*, the drug dosing concentration was set to be relatively low (at which the proliferation rate of sensitive cell a_S was inhibited to half of its value) so that a larger range of the relative proportion of sensitive cells could be examined.

1.3.3 Tumor growth is affected by schedules of treatments.

Our model could clearly describe the growth of two subclones with drug dosing:

$$\frac{dN_R}{dt} = \hat{a}_R(N^{-1} \cdot N_m^{3/4}(t))N_R - \hat{b}_R N_R,$$

$$\frac{dN_S}{dt} = \hat{a}_S(c_{drug})(N^{-1} \cdot N_m^{3/4}(t))N_S - \hat{b}_S N_S.$$

Here, the proliferation rate of sensitive cells $\hat{a}_S(c_{drug})$ became a function of the drug concentration c_{drug}. Generally, the effect of drug could be modeled by a Hill function, i.e., $\hat{a}_S(c_{drug}) = \frac{\hat{a}_S(0)c_{drug}}{C_0+c_{drug}}$. Considering the proliferation of sensitive cells were suppressed by drugs, the fraction of apoptosis sensitive cells would increase, but since we took $\hat{b}_S$ remained unchanged, so $C_0 < IC50$. In our model, c_{drug} was changing over time due to dosing and the drug metabolism. In this way, our model could describe how the growth of tumors would be affected by different schedules of treatments.

During computation, we took a relatively low concentration of the drug c^* so that $\hat{a}_S|_{c^*} = 0.5\hat{a}_S$, i.e, $c^* = C_0$. In such a situation, the drug metabolism could be recognized to be relatively fast due to low drug concentration, which simplified our computation. Thus, c_{drug} could be considered to be mainly determined by a dosing timetable. Besides, a wider range of the fraction of sensitive cells could be examined otherwise the sensitive cells would be fast killed. To model the cases of high concentration of drugs, a lower proliferation rate or/and a higher death rate of sensitive cells should be introduced, and it could be proved that the results would be similar to the similar cases with high dosing frequency at low concentration.

And such control on the proliferation rate of sensitive cells can always be mapped with real dosing process. Mathematically, when the half-time of the drug is τ, and the drug dosed at frequency f_d with concentration c_{drug}, in a long therapy, when the drug is just dosed, the effective concentration $c' = \frac{c_{drug}}{1-\exp(-T_0/f_d \cdot \tau)}$, in which T_0 is a constant related to the course of treatment. Such a result suggests that when dosed with a certain

frequency, the effective concentration of the drug is approximately proportional to the dosing concentration, i.e., our analysis is still meaningful, if we have detailed information about the half-time *in vivo*, our model can be easily generalized to such occasion.

2 Evaluating therapy schedule for different initial conditions

In the main text, we took the $\phi_R^0 = 5\%$ and $V_0 = 1\text{mm}^3$ ($N_0 = 10^6$) case as an example to design the chemotherapeutic schedule. We mentioned in the "Result" and "Discussion" part that our model could also be employed to evaluate the dosing schedule for tumors with different initial fraction of resistant cells ϕ_R^0 and a different initial volume V_0. Here, the details for the evaluation of therapy schedule for different initial conditions were listed.

And we mainly focused on the increase of the patients' survival times (both the ultimate survival time τ_S^{utl} and the expected survival time τ_S^{exp}) with periodic dosing compared with continuous dosing.

2.1 The best dosing frequencies are not sensitive to different initial conditions.

As shown in Supplementary Fig.5 , periodic dosing could increase the patients' ultimate or expected survival time compared with the case of continuous dosing. For different ϕ_R^0 and V_0, the maximum survival time might change significantly, but the best dosing frequencies f_d in order to maximize the ultimate survival time τ_S^{utl} or to maximize the expected survival time τ_S^{exp} remained rather stable. Based on this fact, the best dosing frequency could be easily determined according to different initial conditions. For example, as shown in Supplementary Fig.5, with a larger initial tumor volume ($V_0 > 1\text{mm}^3$) or a larger fraction of resistant cells ($\phi_R^0 > 5\%$), a dosing frequency which was slightly higher than 0.2 would maximize the expected survival time.

2.2 Qualitatively design the therapy strategy for tumors with different initial conditions.

In the main text, the initial condition that we had discussed was a specific case with a small initial tumor volume V_0 and a low initial resistant cell fraction ϕ_R^0. We designed the therapy schedule (switching the dosing frequency f_d between f_d^h and f_d^l) to maintain both the tumor size and the resistant cell fraction at a low level: A high dosing frequency

(e.g., $f_d^h = 0.4$) was employed to reduce the tumor size to a lower level, and a lower dosing frequency ($f_d^l = 0.2$) was employed to maintain the sensitive cells at a larger fraction. Such therapy strategy could be generalized for the tumors with different initial fractions, i.e., one should select f_d^h and f_d^l according to the initial condition of the tumor (ϕ_R^0 and V_0). For example, with a higher ϕ_R^0, intuitively, a lower f_d^l should be applied to inhibit the growth of resistant cells, while with a higher V_0, then one should choose a higher f_d^h to control the tumor size at a low level. Such an intuitive result was also consistent with the simulation result with different initial conditions as shown in Supplementary Fig.5.

References

[1] Marusić, M. et al. (1994). Analysis of growth of multicellular tumor spheroids by mathematical models. *Cell Proliforation*, 27: 7394.

[2] West, Geoffrey B and Brown, James H and Enquist, Brian J. (2001). A general model for ontogenetic growth. *Nature*, 413(6856): 628-631.

[3] West, Geoffrey B and Brown, James H. (2005). The origin of allometric scaling laws in biology from genomes to ecosystems: towards a quantitative unifying theory of biological structure and organization. *Journal of Experimental Biology*, 208(9): 1575-1592.

[4] Guiot, Caterina, et. al. (2003). Does tumor growth follow a "universal law"?. *Journal of Theoretical Biology*, 225(2):147-151.

[5] Rodriguez-Brenes, Ignacio A and Komarova, Natalia L and Wodarz, Dominik. (2013). Tumor growth dynamics: insights into evolutionary processes. *Trends in Ecology & Evolution*, 28(10): 597-604.

[6] Aktipis, C Athena and et. al. (2013). Life history trade-offs in cancer evolution. *Nature Reviews Cancer*, 13(12): 883-892.

[7] Raj L, Ide T, Gurkar AU, Foley M, Schenone M, et al. (2011). Selective killing of cancer cells by a small molecule targeting the stress response to ROS. *Nature*, 475: 231-234.

A Summary of the Mathematical Modeling

I. The growth of a single subclone.

$$\frac{dN_R}{dt} = a_R N_R^{\frac{3}{4}} - b_R N_R,$$ Resistant cells

$$\frac{dN_S}{dt} = a_S N_S^{\frac{3}{4}} - b_S N_S,$$ Sensitive cells

(a) The asymptotic cell numbers of the tumor:

$$N_R^* = \left(\frac{a_R}{b_R}\right)^4 \qquad N_S^* = \left(\frac{a_S}{b_S}\right)^4 \qquad N_R^* = N_S^* = N^*$$

$$V^* = 5000\text{mm}^3 \qquad N^* \sim V^*,$$

And for every cell, $V_{\text{cell}} = 10^{-6}\text{mm}^3$

(b) Different growth rate of resistant and sensitive cells (based on *in vivo* experiments).

$$a_R/a_S \approx 0.35. \qquad a_R = 0.37, a_S = 1.03.$$

II. The growth of multiple subclones.

$$\frac{dN}{dt} = \hat{a}N^{\frac{3}{4}} - \hat{b}N. \qquad (\hat{a} \neq a_S \text{ and } \hat{a} \neq a_R).$$

The growth equation for the two subclones:

$$\frac{dN_R}{dt} = \hat{a}_R N^{-\frac{1}{4}} N_R - \hat{b}_R N_R,$$ Resistant cells

$$\frac{dN_S}{dt} = \hat{a}_S N^{-\frac{1}{4}} N_S - \hat{b}_S N_S.$$ Sensitive cells

(a) Tumor composition:

$$N = N_R + N_S.$$

$\phi_S = N_S/N$, and $\phi_R = N_R/N = 1 - \phi_S.$

(b) The asymptotic volume of the tumor.

$$N_S^* = N_R^* = N^* = (a_R/b_R)^4 = (a_S/b_S)^4. \qquad V^* = 5000 \text{ mm}^3$$

(c) The growth rate of tumor depended on the initial fraction of sensitive cells (based on *in vivo* experiments).

$$\hat{a}(\phi_S) = a_R(1 - \phi_S) + a_S\phi_S.$$

(d) The growth rate of sensitive cells. (Dynamic inhibition rate, which is consistent with experiments.)

$$\hat{a}_S \approx \frac{a_S\phi_S^0}{\phi_S}\left(1 - \frac{a_R}{a_S}\right) + a_R,$$

$$\hat{b}_S \approx \frac{b_S\phi_S^0}{\phi_S}\left(1 - \frac{a_R}{a_S}\right) + b_R.$$

* The slow growth of resistant cells.

$$\hat{a}_R \approx a_R \text{ and } \hat{b}_R \approx b_R.$$

III. The growth of multiple subclones with drugs.

The growth equation for the two subclones:

$$\frac{dN_R}{dt} = \hat{a}_R(N^{-1} \cdot N_m^{3/4})N_R - \hat{b}_R N_R,$$ Resistant cells

$$\frac{dN_S}{dt} = \hat{a}_S(c_{drug})(N^{-1} \cdot N_m^{3/4})N_S - \hat{b}_S N_S.$$ Sensitive cells

Summing up, $\frac{dN}{dt} = \hat{a}N^{\frac{3}{4}} - \hat{b}N. \qquad (\hat{a} \neq a_S \text{ and } \hat{a} \neq a_R).$

(a) The growth rate of sensitive cells. (Without drugs.)

$$\hat{a}_S \approx \frac{a_S\phi_S^0}{\phi_S}\left(1 - \frac{a_R}{a_S}\right) + a_R,$$

$$\hat{b}_S \approx \frac{b_S\phi_S^0}{\phi_S}\left(1 - \frac{a_R}{a_S}\right) + b_R.$$

(b) The effect of the drugs.

$$\hat{a}_S(c_{drug}) = \frac{\hat{a}_S(0)c_{drug}}{C_0 + c_{drug}}.$$

Simplification, $\hat{a}_S|_{c^*} = 0.5\hat{a}_S$, i.e, $c^* = C_0$.

(c) The total amount of vessels was not reduced after treatment.

$N_m(t)$ was the maximum number of total cancer cells during the growth history from the initial time to time t.

$N^{-1} \cdot N_m(t)^{3/4}$ could be estimated as the amount of vessels per cell.

IV. Model Evaluation.

- Dosing frequency f_d as the fraction of time (in unit of days) with the growth of sensitive cells suppressed in a course of treatment (15 days).

(1) Ultimate survival time τ_S^{ult} as the time it took for the tumor grew to a mortal level V^{ult} ($V^{ult} = 4000 \text{ mm}^3$)

(2) Expected survival time τ_S^{exp} as the days during which the tumor size could be controlled below a safe threshold V^{exp} by therapy ($V^{exp} = 3000 \text{ mm}^3$).

(3) Average tumor burden $\langle V \rangle$.
